# Increased proliferation is associated with CNS invasion in meningiomas

**DOI:** 10.1007/s11060-021-03892-7

**Published:** 2021-11-20

**Authors:** Felix Behling, Christina Fodi, Sophie Wang, Johann-Martin Hempel, Elgin Hoffmann, Ghazaleh Tabatabai, Jürgen Honegger, Marcos Tatagiba, Jens Schittenhelm, Marco Skardelly

**Affiliations:** 1 Department of Neurosurgery, University Hospital Tübingen, Eberhard-Karls-University Tübingen, Tübingen, Germany; 2 Department of Diagnostic and Interventional Neuroradiology, University Hospital Tübingen, Eberhard-Karls-University Tübingen, Tübingen, Germany; 3 Department of Radiation-Oncology, University Hospital Tübingen, Eberhard-Karls-University Tübingen, Tübingen, Germany; 4 Department of Neurology & Interdisciplinary Neuro-Oncology, University Hospital Tübingen, Eberhard-Karls-University Tübingen, Tübingen, Germany; 5 Hertie Institute for Clinical Brain Research, Tübingen, Germany; 6 Center for CNS Tumors, Comprehensive Cancer Center Tübingen-Stuttgart, University Hospital Tübingen, Eberhard-Karls-University Tübingen, Tübingen, Germany; 7 German Cancer Consortium (DKTK), DKFZ partner site Tübingen, Tübingen, Germany; 8 Department of Neuropathology, University Hospital Tübingen, Eberhard-Karls-University Tübingen, Tübingen, Germany

**Keywords:** Meningioma, CNS invasion, Brain invasion, MIB1, Ki67, Proliferation

## Abstract

**Introduction:**

Meningiomas are the most common benign intracranial neoplasms. CNS invasion in meningiomas has been integrated into the 2016 WHO classification of CNS tumors as a stand-alone criterion for atypia. Since then, its prognostic impact has been debated based on contradictory results from retrospective analyses. The aim of the study was to elucidate whether histopathological evidence of CNS invasion is associated with increased proliferative potential.

**Methods:**

We have conducted a quantified measurement of the proliferation marker Ki67 and analyzed its association with CNS invasion determined by histology together with other established prognostic markers of progression. Routine, immunohistochemical staining for Ki67 were digitalized and automatic quantification was done using Image J software.

**Results:**

Overall, 1718 meningiomas were assessed. Histopathological CNS invasion was seen in 108 cases (6.7%). Uni- and multivariate analysis revealed a significantly higher Ki67 proliferation rate in meningiomas with CNS invasion (p < 0.0001 and p = 0.0098, respectively).

**Conclusions:**

Meningiomas with histopathological CNS invasion show a higher proliferative activity.

**Supplementary Information:**

The online version contains supplementary material available at 10.1007/s11060-021-03892-7.

## Introduction

Meningioma is the most common benign tumor of the central nervous system and makes up one third of primary intracranial tumors [1]. These tumors are usually slow growing and arise from the arachnoid cap cells of the meninges [2]. Treatment by microsurgical excision is sufficient for curing most patients, while radiation therapy is reserved for selected and recurrent cases [3]. About 20% of meningiomas recur [4] and some sources claim an even higher recurrence rate of up to 47% with a long follow-up of 25 years [5]. Therefore, it is of great importance to identify patients with an increased risk of meningioma recurrence to guide postoperative management. Besides the long-established histopathological assessment according to the WHO classification of central nervous system tumors [4], the detection of infiltrative meningioma growth into brain parenchyma has been added as a stand-alone criterion for atypia [4]. However, its prognostic significance has since been questioned based on contradictory results of retrospective analyses [6–9] and its role for tumor grading in the WHO classification is frequently discussed [10, 11].

We have recently compared the prognostic role of the histopathological and intraoperative detection of CNS invasion in a multivariate model in a large meningioma cohort. While each detection by itself did not reach prognostic significance in the multivariate analysis, the combination of both methods did [8]. The reasons for the conflicting evidence of CNS invasion in meningioma are most likely the unstandardized sampling and non-uniform histopathological criteria applied [8, 11]. Before abandoning CNS invasion for meningioma risk stratification prematurely, we believe it is important to keep up interdisciplinary efforts to generate more evidence in this field.

The mentioned retrospective studies that investigated the role of CNS invasion in meningiomas have focused on tumor recurrence as an outcome variable [6–9]. To our knowledge, there has been no detailed analysis of the proliferative activity in meningiomas with histopathological features of infiltrative growth. We have therefore applied a quantification analysis of the immunohistochemical expression of the proliferation marker Ki67 in our meningioma cohort to investigate a possible association of proliferation and infiltrative growth in meningioma.

## Methods

**Fig. 1:**
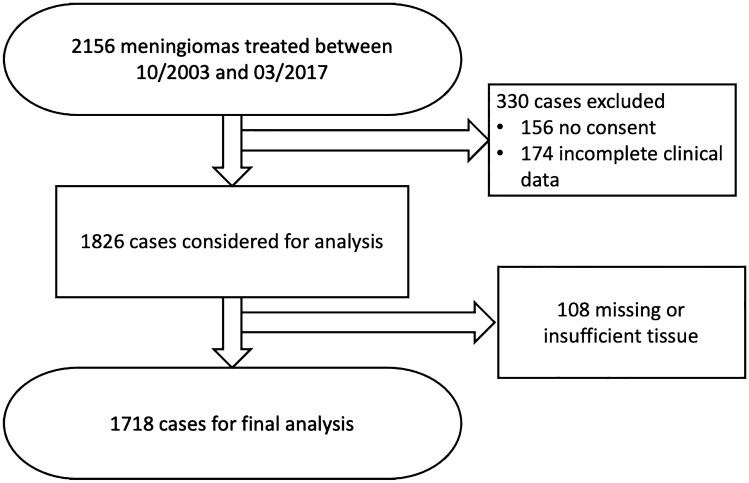
Flow chart of the composition of the study cohort

In this single center retrospective analysis, we analyzed the histopathological CNS invasion and other clinical factors regarding its association with the immunohistochemical expression of the proliferation marker Ki67 in a large cohort of meningiomas. Overall, 2156 meningiomas were surgically treated in the authors’ institution between October 2003 and March 2017. 330 cases with missing consent for scientific utilization, incomplete clinical data and 108 cases with missing or poor-quality tissue were excluded (Fig. 1).

The following clinical factors were collected for all included cases via a systematic review of available clinical documents and radiographic imaging: age at diagnosis, gender, tumor status (primary/recurrent), radiotherapy prior to surgery, diagnosis of neurofibromatosis type 2, tumor location, extent of resection (according to the Simpson classification [12]). In the authors’ institution, CNS invasion was determined based on the histologic criteria defined by Perry [13]. In that regard, presence of irregular protrusions of meningioma cells into CNS parenchyma without a leptomeningeal layer in between was defined as invasive growth. On the other hand, perivascular spread into Virchow-Robin spaces was not graded as such. Histopathological reports were reviewed and cases with clearly stated CNS invasion identified. If no statement regarding CNS invasion was documented, cases were graded as non-invasive. To analyze CNS invasion as an independent co-factor, brain-invasive but otherwise benign meningiomas were graded as outlined by the WHO classification of 2007 as WHO grade I [14], since it does not incorporate CNS invasion as sole grading criterion for atypia in comparison to the current classification of 2016 [4].

**Fig. 2:**
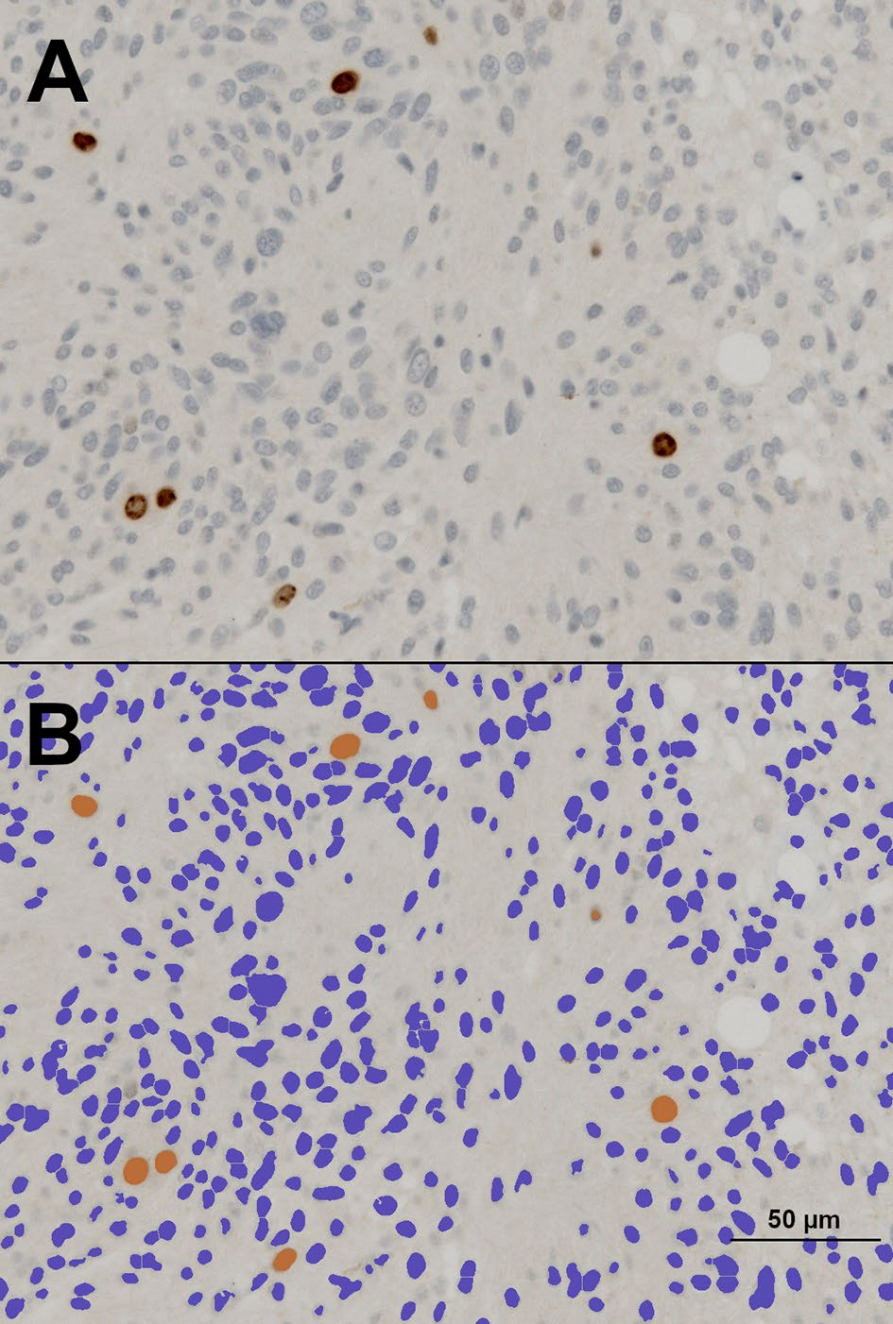
Example of the digital quantification of the immunohistochemical expression of Ki67 in tumor nuclei. Panel **A** shows the Ki67 diaminobenzidine staining (brown chromogen) and negative tumor cells counterstained with hematoxylin (blue) and panel **B** the corresponding quantitative computer-assisted measurement (400-fold magnification)

Immunohistochemical stainings for Ki67, that were routinely prepared during the histopathological diagnostic process, were retrieved and quantitatively reassessed. Digital images were taken of representative areas of each Ki67 staining and quantitative measurements of areas of immunopositivity were done with the Image J software (Version 1.51j8, NIH, Bethesda, MD, USA) and the plugins Bio-Formats (Release 5.4.1; Open Microscopy Environment, Madison, NJ, USA) and ImmunoRatio (Version 1.0c, Institute of Biomedical Technology, University of Tampere, Finland). Corresponding images that highlighted nuclei detected as stained were generated and matched with original stains to ensure quantification values consistent with the neuropathological assessment (Fig. 2).

For statistical analysis the JMP® Statistical Discovery Software was used (Version 15.1.0, Cary, NC: SAS Institute Inc.; 1989). Univariate analysis of clinical and histopathological factors regarding differences in Ki67 expression was done with ANOVA and a linear regression was done for multivariate analysis. The level of significance was set at α < 0.05. Factors that showed significant results in the univariate analysis were included in the multivariate analysis.

## Results

### Cohort characteristics

Overall, 1718 meningiomas were included for further analysis, consisting of 1229 female and 489 male patients (female to male ratio 2.51). The mean age of the cohort was 57.23 years, ranging from 3.83 to 90.96 years. The majority of meningiomas were primary tumors (n = 1504, 87.5%) while 214 cases were recurrent tumors (12.5%). Eighty meningiomas received radiotherapy prior to surgical resection (4.7%). One-hundred and three tumors were from patients suffering from neurofibromatosis type 2 (6.0%). The tumor location was categorized into convexity/falx (n = 649, 37.8%), skull base (n = 893, 52%) and spinal (n = 176, 10.2%). According to the WHO classification of central nervous system tumors from 2007, 1412 meningiomas were graded as WHO grade I (82.2%), 285 as grade II (16.6%) and 21 as grade III (1.2%). CNS invasion was histopathologically detected in 108 cases (6.7%) (for details see Table 1). The distribution of all analyzed factors and their interrelations are displayed in the Supplementary Table 1.

**Table 1:** Cohort characteristics and Ki67 expression

Variable	n(%)	Ki67 expression (% immunopositive)	p-value (ANOVA)	p-value (linear regression)
Gender				
F	1229 (71.5)	2.64	< .0001*	0.0014*
M	489 (28.5)	3.77		
Age			0.0125*	0.3385
≥ 70.5	353 (20.5)	3.31		
< 70.5	1365 (79.5)	2.87		
Tumor status			< .0001*	< .0001*
Primary	1504 (87.5)	2.62		
Recurrent	214 (12.5)	5.36		
Prior RT			< .0001*	< .0001*
Yes	80 (4.7)	7.68		
No	1638 (95.3)	2.73		
Neurofibromatosis type 2			0.3007	
Yes	103 (6.0)	2.67		
No	1615 (94.0)	2.98		
Tumor location			< .0001*	0.0002*
Convexity/Falx	649 (37.8)	3.60		
Skull base	893 (52.0)	2.54		
Spinal	176 (10.2)	2.77		
WHO classification of 2007			< .0001*	< .0001*
I	1412 (82.2)	2.42		
II	285 (16.6)	4.99		
III	21 (1.2)	12.14		
CNS invasion			< .0001*	0.0098*
Yes	108 (6.7)	5.33		
No	1610 (93.7)	2.81		

*ANOVA* analysis of variance; *CNS* central nervous system; *RT* radiotherapy; *WHO* World Health Organization

Asterisks(*) mark statistically significant results

### Univariate analysis

**Fig. 3:**
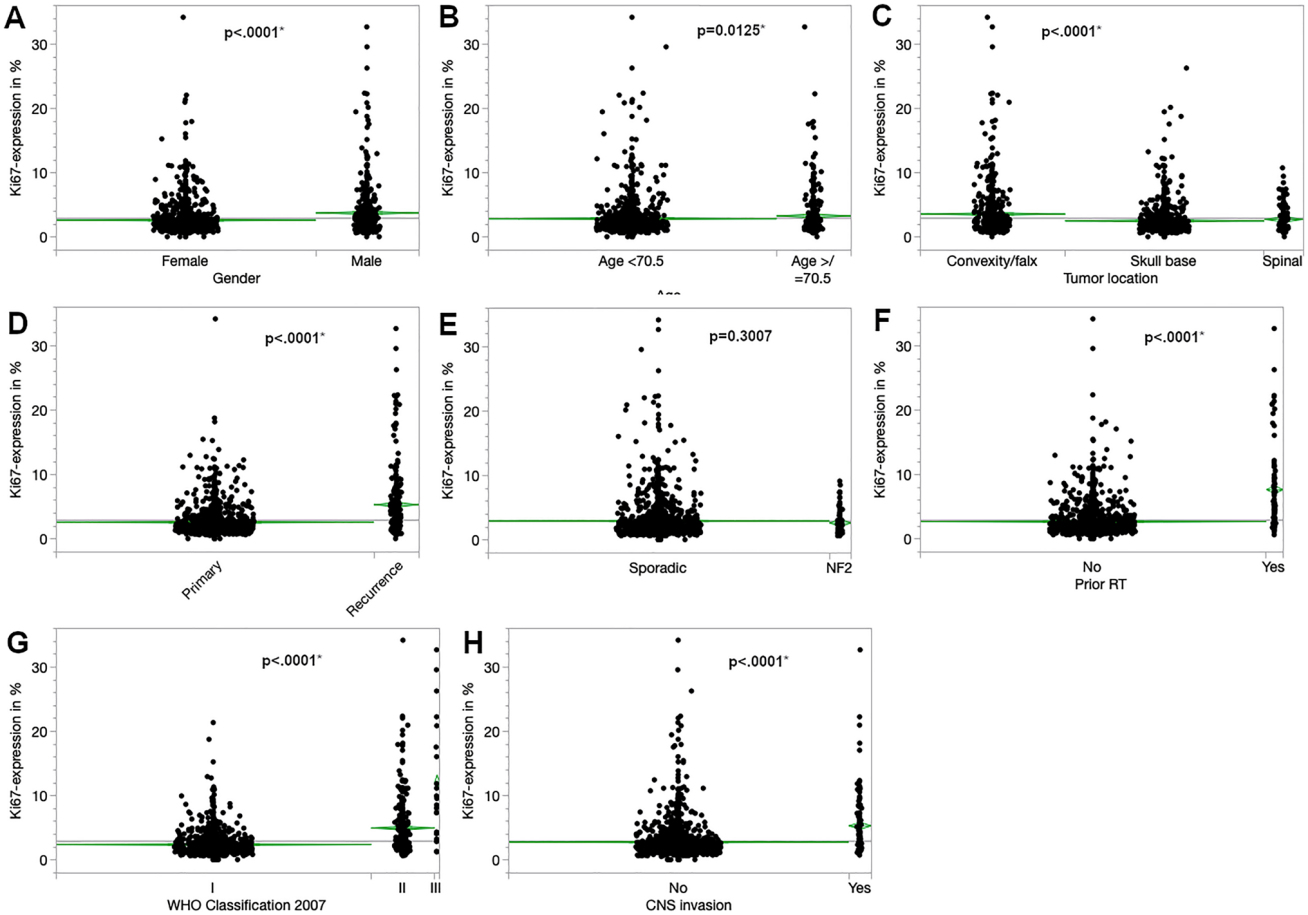
Univariate analysis of the immunohistochemical expression of Ki67 according to gender (**A**), age (**B**), tumor location (**C**), tumor status (**D**), neurofibromatosis type 2 (**E**), prior radiotherapy (**F**), WHO classification 2007 (**G**) and CNS invasion (**H**). Asterisks (*) mark statistically significant results

Meningiomas in male patients showed a significant higher Ki67 expression than in females (3.77% and 2.64%, p < 0.0001). Regarding the influence of age, the largest difference of Ki67 expression was seen with a cutoff at 70.5 years of age according to a CART analysis. Patients with an age equal or above 70.5 years had a mean Ki67 expression of 3.31% compared to 2.87% for younger patients (p = 0.0125). Recurrent tumors showed a higher rate of immunopositivity for Ki67 as compared to primary meningiomas (5.36% compared to 2.62%, p < 0.0001). Similarly, increased Ki67 immunostaining was found in patients with prior radiotherapy (7.68% with radiotherapy compared to 2.73% without radiotherapy, p < 0.0001). Within recurrent meningiomas this difference was quite similar (n = 214); cases that had received prior radiotherapy also had a higher Ki67 score than non-irradiated recurrent tumors (7.9% vs. 3.9%, p < 0.0001). There was no significant difference in Ki67 expression for NF2 patients. Meningiomas located at the convexity or falx had the highest immunopositivity (3.60%), followed by spinal tumors (2.77%) and the lowest rate was seen for skull base meningiomas (2.54%, p < 0.0001). With increasing WHO grade (WHO classification of 2007), higher mean Ki67 expression scores were seen (WHO grade I: 2.42%, WHO grade II: 4.99% and WHO grade III: 12.14%, p < 0.0001). Meningiomas with CNS invasion showed almost a double mean immunopositivity for the proliferation marker (5.33% compared to 2.81%, p < 0.0001). Details of the univariate analysis are displayed in Fig. 3 and Table 1.

### Multivariate analysis

All factors that showed significant associations with Ki67 expression in the univariate analysis were integrated into the multivariate linear regression. Presence of histopathological CNS invasion was an independent factor for increased Ki67 expression rates (p = 0.0098). Furthermore, male gender, recurrent tumor status, higher WHO grade according to the classification of 2007, prior radiotherapy and convexity/falx tumor location were also independently associated with higher proliferation marker values (Details are shown in Table 1).

## Discussion

The clinical impact of CNS invasion in meningioma is increasingly critically discussed since its integration into the WHO classification for CNS tumors in 2016 [4, 11]. The current knowledge is primarily based on multiple retrospective analyses that found no prognostic impact of CNS invasion [7, 9, 15]. However, it is important to keep in mind that two issues with the detection of CNS invasion exist, that have likely impacted retrospective studies. First of all, the histopathological characteristics used to determine infiltrative growth are not clearly defined [10, 11] and possibly vary between departments and neurooncological centers. Additionally, intraoperative tumor sampling is non-standardized and especially areas of interest may not always be amenable to appropriate sampling [16]. We recently compared the prognostic potential of histopathological and intraoperative detection of infiltrative growth in 1517 meningiomas. We found that both methods do not show an independent prognostic impact by itself, as they are currently applied, but if they are combined. Our findings underlined the need to further assess the prognostic impact by other methods and to investigate the histopathological and intraoperative detection of CNS invasion in a prospective and controlled fashion [8].

The tumor cell proliferation rate is an integral part of the WHO classification for CNS tumors. The assessment of the mitotic index by detection of mitoses per 10 high-power fields is an established measure when considering the diagnosis of atypical or anaplastic meningioma [2]. Immunohistochemical expression of Ki67 as direct visualization of proliferating cells [17], is not a criterion for pathological grading but has long been suggested as a prognostic marker in meningioma [18] and the increased risk of tumor recurrence in WHO grade I meningiomas based on increased Ki67 expression has recently been demonstrated [19]. Another recent study evaluated a preoperative scoring system based on sex, peritumoral edema, preoperative CRP value, and plasma fibrinogen level and found a Ki67 cutoff of > 6% prognostic to predict tumor recurrence but the cohort was not stratified for brain infiltration [20].

However, variations in interobserver interpretation and different staining protocols make it difficult to establish clear cut off values. The consideration of the mitotic index for prognostic assessment is still essential for tumor grading in the upcoming WHO classification while inclusion of further proliferation quantification has been recommended by some authors [2, 21]. In a recent study the digital assessment of Ki67 immunohistochemistry demonstrated a good correlation with manual determination in 141 meningiomas [22]. We have therefore used a computerized quantification method to control for an interobserver bias and to obtain continual numerical values. We have recently demonstrated the independent significant prognostic impact of quantified Ki67 expression in our meningioma cohort [23]. However, it must be kept in mind, that Ki67 positivity may also include proliferating inflammatory cells and therefore may suggest falsely increased proliferation characteristics. An increased lymphocytic infiltrate may be due to higher WHO grade [24] and could also be associated with prior radiotherapy. This is the main limitation of the method used in our study and hopefully future studies will provide robust data on that inaccuracy to further refine this unique method. Another potential issue that needs to be mentioned is that the applied characteristics for the assessment of invasive growth into CNS tissue may vary in different centers. Our institution has routinely applied the widely used criteria defined by Perry in 1997 [13] in the histopathological meningioma work-up that makes up this cohort. It has been described that the assessment of pathology concordance in the NRG Oncology RTOG Trial 0539 showed an especially high agreement for brain invasion with 92.4%. Furthermore, the evaluation for >  = 4 mitoses per 10 high power fields, which is one of the most important diagnostic criteria for atypia in meningiomas, had one of the lowest levels of concordance (79.1%) [25].

The pathophysiology of invasive tumor growth of meningiomas is still unknown. Cell-to-cell contact has long been established as an important factor for the suppression of proliferation in cancer [26]. A crucial role has been attributed to merlin, a protein which is absent in many meningiomas due to *NF2* loss. Merlin is known to mediate contact inhibition of mitogenic activity by modulating membrane receptor signaling and cadherin-mediated cell-to-cell contact [27]. This suggests a possible association of CNS invasion and proliferation in meningiomas, which may be a factor explaining the results of this study. However, the exact mechanism of invasive growth in meningiomas is yet to be described and possibly aberrations can be revealed that may be therapeutically addressable or diagnostically exploitable.

Furthermore, infiltrative growth into other structures like adjacent bone may have a different pathophysiology. For example, in sphenoid wing meningiomas, varying grades of bone involvement were associated with different genomic profiles. While bone invasion was associated with NF2 mutations, hyperostosis was seen more often together with TRAF7 aberrations [28].

In this study we showed that Ki67 expression is independently associated with histopathological detection of CNS invasion suggesting that meningiomas with infiltrative growth have a higher proliferation rate compared to tumors of the same grade where CNS invasion is absent. To our knowledge, this is the first study to show this relationship. It underlines the prognostic potential of CNS invasion in meningioma. However, if the nature of infiltrative growth is biologically associated with the proliferative activity of meningioma cells, remains unclear. It is possible that another variable like genetic instability may act as confounding factor [29]. The mechanism of CNS invasion may occur independently from tumor cell proliferation. But our data clearly show, that meningiomas that have developed invasive features, have a significant higher proliferative marker expression, and thus can be considered as a more aggressive entity. This supports the decision expressed in the new WHO classification for CNS tumors of 2021, which still incorporates CNS invasion as a stand-alone criterion for atypia [2]. Understandably, the role of infiltrative growth in brain parenchyma is still controversial, especially due to non-standardized sampling and histopathological grading as recently expressed [11, 16]. Our data provide a contribution to this topic, but more robust studies are needed to further our understanding of the mechanism of CNS invasion. This may also reveal targets for specific therapies that could possibly extend the few treatment options currently available, especially for patients with advanced meningiomas, when surgical and radiotherapeutic options have been exhausted.

## Conclusions

Histopathological detection of CNS invasion in meningioma is an independent factor for increased expression of the proliferation marker Ki67, underlining the association of infiltrative growth and proliferative activity.

## Supplementary Information

Below is the link to the electronic supplementary material.

Supplementary file1 (DOCX 23 kb)

## Data Availability

The dataset is available upon reasonable request.

## Code availability

No applicable.
