# Supplementary material for: Increased proliferation is associated with CNS invasion in meningiomas
Source: J Neurooncol. 2021 Nov 20;155(3):247–54. doi: 10.1007/s11060-021-03892-7 (PMC8651603; doi:10.1007/s11060-021-03892-7)
Supplement: Supplementary file 1 — Supplementary file1 (DOCX 23 kb) [file 11060_2021_3892_MOESM1_ESM.docx]

**Supplementary Table 1.** Distribution of all analyzed factors and their interrelations.

|  | **Gender** | | **Age** | | **Tumor status** | | **Prior RT** | | **NF2** | | **Location** | | | **WHO 2007** | | | **CNS invasion** | |
| --- | --- | --- | --- | --- | --- | --- | --- | --- | --- | --- | --- | --- | --- | --- | --- | --- | --- | --- |
|  | **F** | **M** | **>=70.5** | **<70.5** | **Prim** | **Rec** | **Yes** | **No** | **Yes** | **No** | **Convexity/falx** | **Skull base** | **Spinal** | **I** | **II** | **III** | **Yes** | **No** |
| **Gender**  **F**  **M** | 1229 | 489 | 250 (20.3)  103 (21.1) | 979 (79.7)  386 (78.9) | 1099 (89.4)  405 (82.8) | 130 (10.6)  84 (17.2) | 37 (3.0)  43 (8.8) | 1192 (97.0)  446 (91.2) | 54 (4.4)  49 (10.0) | 1175 (95.6)  440 (90.0) | 437 (35.6)  212 (43.4) | 655 (53.3)  238 (48.7) | 137 (11.2)  39 (8.0) | 1073 (87.3)  339 (69.3) | 150 (12.2)  135 (27.6) | 6 (0.5)  15 (3.1) | 57 (4.6)  51 (10.4) | 1172 (95.4)  438 (89.6) |
| **Age**  **>=70.5**  **<70.5** | 250 (70.8)  979 (71.7) | 103 (29.2)  386 (28.3) | 353 | 1365 | 309 (87.5)  1195 (87.6) | 44 (12.5)  170 (12.5) | 19 (5.4)  61 (4.5) | 334 (94.6)  1304 (95.5) | 1 (0.3)  102 (7.5) | 352 (99.7)  1263 (92.5) | 157 (44.5)  492 (36.0) | 134 (38.0)  759 (55.6) | 62 (17.6)  114 (8.4) | 269 (76.2)  1143 (83.7) | 79 (22.4)  206 (15.1) | 5 (1.4)  16 (1.2) | 35 (9.9)  73 (5.4) | 318 (90.1)  1292 (94.7) |
| **Tumor status**  **Prim**  **Rec** | 1099 (73.1)  130 (60.8) | 405 (26.9)  84 (39.3) | 309 (20.6)  44 (20.6) | 1195 (79.5)  170 (79.4) | 1504 | 214 | 4 (0.3)  76 (35.5) | 1500 (99.7)  138 (64.5) | 89 (5.9)  14 (6.5) | 1415 (94.1)  200 (93.5) | 556 (37.0)  93 (43.5) | 780 (51.9)  113 (52.8) | 168 (11.2)  8 (3.7) | 1301 (86.5)  111 (51.9) | 201 (13.4)  84 (39.3) | 2 (0.1)  19 (8.9) | 74 (4.9)  34 (15.9) | 1430 (95.1.)  180 (84.1) |
| **Prior RT**  **Yes**  **No** | 37 (46.3)  1192 (72.8) | 43 (53.8)  446 (27.2) | 19 (23.8)  334 (20.4) | 61 (76.3)  1304 (79.6) | 4 (5.0)  1500 (91.6) | 76 (95.0)  138 (8.4) | 80 | 1638 | 2 (2.5)  101 (6.2) | 78 (97.5)  1537 (93.8) | 38 (47.5)  611 (37.3) | 41 (51.3)  852 (52.0) | 1 (1.3)  175 (10.7) | 24 (30.0)  1388 (84.7) | 39 (48.8)  246 (15.0) | 17 (21.3)  4 (0.2) | 21 (26.3)  87 (5.3) | 59 (73.8)  1551 (94.7) |
| **NF2**  **Yes**  **No** | 54 (52.4)  1175 (72.8) | 49 (47.6)  440 (27.2) | 1 (1.0)  352 (21.8) | 102 (99.0)  1263 (78.2) | 89 (86.4)  1415 (87.6) | 14 (13.6)  200 (12.4) | 2 (1.9)  78 (4.8) | 101 (98.1)  1537 (95.2) | 103 | 1615 | 35 (34.0)  614 (38.0) | 53 (51.5)  840 (52.0) | 15 (14.6)  161 (10.0) | 83 (80.6)  1329 (82.3) | 20 (19.4)  265 (16.4) | 0 (-)  21 (1.3) | 5 (4.9)  103 (6.4) | 98 (95.2)  1512 (93.6) |
| **Location**  **Convexity/falx**  **Skull base**  **Spinal** | 437 (67.3)  655 (73.4)  137 (77.8) | 212 (32.7)  238 (26.7)  39 (22.2) | 157 (24.2)  134 (15.0)  62 (35.2) | 492 (75.8)  759 (85.0)  114 (64.8) | 556 (85.7)  780 (87.4)  168 (95.5) | 93 (14.3)  113 (12.7)  8 (4.6) | 38 (5.9)  41 (4.6)  1 (0.6) | 611 (94.1)  852 (95.4)  175 (99.4) | 35 (5.4)  53 (5.9)  15 (8.5) | 614 (94.6)  840 (94.1)  161 (91.5) | 649 | 893 | 176 | 450 (69.3)  792 (88.7)  170 (96.6) | 186 (28.7)  94 (10.5)  5 (2.8) | 13 (2.0)  7 (0.8)  1 (0.6) | 75 (11.6)  33 (3.7)  0 (-) | 574 (88.4)  860 (96.3)  176 (100) |
| **WHO 2007**  **I**  **II**  **III** | 1073 (76.0)  150 (52.6)  6 (28.6) | 339 (24.0)  135 (47.4)  15 (71.4) | 269 (19.1)  79 (27.7)  5 (23.8) | 1143 (81.0)  206 (72.3)  16 (76.2) | 1301 (92.1)  201 (70.5)  2 (9.5) | 111 (7.9)  84 (29.5)  19 (90.5) | 24 (1.7)  39 (13.7)  17 (81.0) | 1388 (98.3)  246 (86.3)  4 (19.1) | 83 (5.9)  20 (7.0)  0 (-) | 1329 (94.1)  265 (93.0)  21 (100) | 450 (31.9)  186 (65.3)  13 (61.9) | 792 (56.1)  94 (33.0)  7 (33.3) | 170 (12.0)  5 (1.8)  1 (4.8) | 1412 | 285 | 21 | 35 (2.5)  68 (23.9)  5 (23.8) | 1377 (97.5)  217 (76.1)  16 (76.2) |
| **CNS invasion**  **Yes**  **No** | 57 (52.8)  1172 (72.8) | 51 (47.2)  438 (27.2) | 35 (32.4)  318 (19.8) | 73 (67.6)  1292 (80.3) | 74 (68.5)  1430 (88.8) | 34 (31.5)  180 (11.2) | 21 (19.4)  59 (3.7) | 87 (80.6)  1551 (96.3) | 5 (4.6)  98 (6.1) | 103 (95.4)  1512 (93.9) | 75 (69.4)  574 (35.7) | 33 (30.6)  860 (53.4) | 0 (-)  176 (10.9) | 35 (32.4)  1377 (85.5) | 68 (63.0)  217 (13.5) | 5 (4.6)  16 (1.0) | 108 | 1610 |
